# Daptomycin and methicillin-resistant *Staphylococcus aureus* isolated from a catheter-related bloodstream infection: a case report

**DOI:** 10.1186/1756-0500-7-759

**Published:** 2014-10-25

**Authors:** Fernanda S Cavalcante, Dennis de C Ferreira, Raiane C Chamon, Thaina M da Costa, Fernanda Maia, Elaine M Barros, Tatiana Silva Dantas, Kátia R N dos Santos

**Affiliations:** Departamento de Microbiologia Médica, Laboratório de Infecção Hospitalar/UFRJ, Instituto de Microbiologia Paulo de Góes, Universidade Federal do Rio de Janeiro, Av. Carlos Chagas Filho, 373, CEP: 21941–590 Rio de Janeiro, Brazil; Escola de Odontologia, Universidade Veiga de Almeida, Rio de Janeiro, Brazil; Hospital Naval Marcilio Dias, Rio de Janeiro, Brazil; Faculdade de Ciências Farmacêuticas, Universidade de São Paulo, São Paulo, Brazil

**Keywords:** MRSA, Daptomycin, Catheter-related bloodstream-infection, SCC*mec* II

## Abstract

**Background:**

Daptomycin is an alternative option for the treatment of catheter-related bloodstream-infections caused by methicillin-resistant *Staphylococcus aureus*. This study reports a case of a daptomycin and methicillin-resistant *Staphylococcus aureus* isolate recovered from the blood of a Brazilian patient undergoing hemodialysis.

**Case presentation:**

A 64-year-old white male patient suffering from diabetes mellitus, systolic hypertension, heart disease with a coronary stent, obesity and chronic renal failure and on use of permcath catheter developed a catheter-related bloodstream-infection by a daptomycin-methicillin-resistant *Staphylococcus aureus* isolate after one month of daptomycin therapy. The isolate was identified as the SCC*mec* II/USA100/sequence type 5 lineage by molecular techniques.

**Conclusions:**

In this work we described a Brazilian patient with bloodstream infection caused by a daptomycin and methicillin-resistant *Staphylococcus aureus* belonging to the lineage USA100/sequence type 5. Our case highlights the careful management of bloodstream infections and the importance of the judicious use of antimicrobials due the possibility of daptomycin-resistance developing among *S. aureus* isolates, especially in patients under hemodialysis, which are frequently exposed to vancomycin and daptomycin therapy.

## Background

Methicillin-resistant *Staphylococcus aureus* (MRSA) is one of the most important causes of catheter-related bloodstream-infections (CRBSI) among hemodialysis patients [1, 2] and daptomycin is an alternative option for the treatment [3]. In Brazil, there is only one report describing two isolates of daptomycin-resistant *S. aureus*; however no information was given concerning the clinical conditions of the patients [4]. This study reports the case of a daptomycin-resistant MRSA isolate recovered from the blood of a Brazilian patient undergoing hemodialysis, also the susceptibility profile and molecular characteristics of the isolate are described.

## Case presentation

A 64-year-old white male, resident in Rio de Janeiro, has been managing our hospital due to insulin dependent diabetes mellitus, arterial hypertension, chronic coronary disease and renal failure. In August of 2011, he began hemodialysis in our hospital as his renal glomerular function became worse. In March 2012, one month after implanting a permcath catheter he came for the emergency department with signs of infection to elucidate. The results of the microscopic examination of urinary sediment showed 15 to 20 white blood cells (WBC), and so cephalexin was begun to treat a possible urinary infection. However, three days after the antibiotic therapy was begun the urinary sample did not show any bacterial growth. The patient returned with signs of systemic inflammatory response syndrome. A clinical examination of the patient showed an inflammation at the insertion site of the hemodialysis catheter. The patient was admitted in our critical care unit on 7 March 2012 with suspicion of CRBSI. The catheter was removed and another was implanted in his right femoral vein. Two samples of peripheral blood were sent for culture and empiric therapy with vancomycin and piperacillin-tazobactam was initiated within one hour of admittance to the ICU. The microbiology analysis of blood samples revealed MRSA. The therapy was changed to daptomycin on 12 March 2012. After 30 doses of daptomycin intravenously based on his glomerular filtrate rate (GFR), the patient was discharged from the hospital on 18 April.

On 9 May 2012, 20 days after the last dose of daptomycin, he returned to the hemodialysis department with a respiratory syndrome. The chest computed tomography demonstrated signs of pneumonia. Therefore, three samples of peripheral blood were sent to the hospital laboratory. Antibiotic therapy (vancomycin, meropenem and trimethoprim-sulfamethoxazole) was initiated according to the microbiology data. The vascular catheter was withdrawn because there was free purulent secretion at the insertion site. Once again, a new catheter was implanted, this time, in the right femoral vein for hemodialysis therapy.

The new MRSA isolate from the blood samples showed a multidrug resistance pattern. The isolate was resistant to ciprofloxacin, rifampin, chloramphenicol, clindamycin and erythromycin but showed susceptibility to linezolid, quinupristin-dalfopristin, tetracycline and trimethoprim-sulfamethoxazole by the disk-diffusion test. The minimum inhibitory concentration (MIC) assessed by broth microdilution method showed MICs of >256 μg/mL, 4 μg/mL and 8 μg/mL for oxacillin, vancomycin and daptomycin, respectively. The SCC*mec* typing [5], pulsed field gel electrophoresis [6] and multilocus sequence typing (MLST) [7] revealed that the isolate belonged to the SCC*mec* II/USA100/ST5 lineage. After 3 months in hospital, the patient died from a hemorrhagic stroke and pneumonia associated mechanical ventilation due to *Acinetobacter baumanni*.

## Discussion

Methicillin-resistant *S. aureus* (MRSA) infections are becoming more frequent and are less easily treated with the current antibiotic agents. There is a high incidence of catheter-related bloodstream-infections (CRBSI) caused by MRSA among patients undergoing hemodialysis and the mortality rates are also high [1, 2]. Although vancomycin is the mainstay treatment for MRSA complicated infections, the experts observed failure of vancomycin therapy associated to high mortality rates have been observed when MRSA isolates exhibits MIC ≥2 μg/mL [8]. According to Clinical Practice Guidelines for the Diagnosis and Management of CRBSI daptomycin is recommended when MRSA isolates exhibit vancomycin MIC >2 μg/mL . In this study, the MRSA isolate recovered from the blood of the patient had a MIC =4 μg/ml to vancomycin, justifying the antibiotic therapy of daptomycin.

Clinical cases have shown decreasing susceptibility of *S. aureus* to daptomycin due to antibiotic pressure exerted by vancomycin, and vice versa [9, 10]. Data from 30 MRSA isolates recovered from bloodstream infections between 2011 and 2013 were evaluated by our group. The results showed that among 13 isolates with a vancomycin MIC =1 μg/ml, 85% showed daptomycin MIC ≥1 μg/ml. Likewise, among 16 isolates with vancomycin MIC ≥2 μg/ml 43.7% also presented MIC ≥2 μg/ml for daptomycin (Table 1; data not published). Kirby *et al*. [11] reported an infection by daptomycin-resistance *S. aureus* from a catheter-related BSI in a hemodialysis patient. The authors confirmed that the MIC to daptomycin of this isolate increased from 0.25 to 0.5 after nine months of various schemes of vancomycin therapy, even though the patient had never received daptomycin. After five months of daptomycin therapy be started, the MIC value to this drug increased to 62 μg/mL. In our case, after 30 doses of daptomycin, the resistance to this drug was detected in the MRSA with MIC 4 μg/mL for vancomycin.

**Table 1:** **Minimum inhibitory concentrations (MIC) of daptomycin of 30 methicillin-resistant** ***S. aureus*** **isolates according to MIC of vancomycin presented by these isolates**

MIC of vancomycin (N° of isolates)	MIC of daptomycin N^o^(%) of isolates			
	0.5	1	2	4
0.5 (1)	1 (100)	0	0	0
1 (13)	2 (15.4)	7 (53.8)	3 (23.1)	1 (7.7)
2 (12)	0	7 (58.3)	4 (3.3)	1 (8.4)
4 (4)	1 (25)	1 (25)	1 (25)	1 (25)

Nevertheless, resistance to daptomycin in *S. aureus* isolates remains rare. A study conducted by a surveillance program analyzed 49,000 *S. aureus* isolates collected from around the world, between 2005 and 2008, and found only 53 (0.05%) daptomycin-resistant isolates. Only one was recovered in Latin American. However, Dabul & Camargo [4] recently reported two daptomycin-resistant *S. aureus* isolates in Brazil, collected from a catheter tip and a leg abscess in different patients. Both the isolates were methicillin-resistant and belonged to the SCC*mec* II/USA100/ST5 lineage, the same profile found in this study. This lineage has established itself in Brazilian hospitals [12, 13]. The genetic similarity found between the isolates indicates a facility of this genotype to develop resistance to daptomycin.

## Conclusion

Vancomycin and daptomycin resistance in *S. aureus* are especially worrying in hemodialysis patients due to the high incidence of CRBSI [1, 2] which are frequently treated with these antimicrobials. In this study we described the CRBSI of a Brazilian patient caused by a daptomycin-resistant MRSA belonging to a lineage already involved in daptomycin-resistance in Brazil. Thus, our case highlights the careful management of BSI infections and the importance of the judicious use of antimicrobials due the possibility of daptomycin-resistance developing among *S. aureus* isolates.

## Consent

Written informed consent was obtained from the patient’s spouse for publication of this Case Report and any accompanying images. A copy of the written consent is available for review by the Editor-in-Chief of BMC Research Notes.

## Abbreviations

SCC*mec*: Staphylococcal chromosome cassette *mec*
ST: Sequence type
MRSA: Methicillin-resistant *S. aureus*
CRBSI: Catheter-related bloodstream-infections
WBC: White blood cells
GFR: Glomerular filtration renal
MIC: Minimum inhibitory concentration
MLST: Multilocus sequence typing.
